# Material Analysis of Coated Siliconized Silicon Carbide (SiSiC) Honeycomb Structures for Thermochemical Hydrogen Production

**DOI:** 10.3390/ma6020421

**Published:** 2013-01-31

**Authors:** Martina Neises-von Puttkamer, Heike Simon, Martin Schmücker, Martin Roeb, Christian Sattler, Robert Pitz-Paal

**Affiliations:** 1 Institute of Solar Research, German Aerospace Center (DLR), Linder Hoehe, Köln 51170, Germany; E-Mails: martin.roeb@dlr.de (M.R.); christian.sattler@dlr.de (C.S.); robert.pitz-paal@dlr.de (R.P.-P.); 2 Institute of Materials Research, German Aerospace Center (DLR), Linder Hoehe, Köln 51170, Germany; E-Mails: heike.simon@dlr.de (H.S.); martin.schmuecker@dlr.de (M.S.)

**Keywords:** thermochemical cycle, water splitting, mixed iron oxides, ferrite, hydrogen, silicon carbide

## Abstract

In the present work, thermochemical water splitting with siliconized silicon carbide (SiSiC) honeycombs coated with a zinc ferrite redox material was investigated. The small scale coated monoliths were tested in a laboratory test-rig and characterized by X-ray diffractometry (XRD) and Scanning Electron Microscopy (SEM) with corresponding micro analysis after testing in order to characterize the changes in morphology and composition. Comparison of several treated monoliths revealed the formation of various reaction products such as SiO_2_, zircon (ZrSiO_4_), iron silicide (FeSi) and hercynite (FeAl_2_O_4_) indicating the occurrence of various side reactions between the different phases of the coating as well as between the coating and the SiSiC substrate. The investigations showed that the ferrite is mainly reduced through reaction with silicon (Si), which is present in the SiSiC matrix, and silicon carbide (SiC). These results led to the formulation of a new redox mechanism for this system in which Zn-ferrite is reduced through Si forming silicon dioxide (SiO_2_) and through SiC forming SiO_2_ and carbon monoxide. A decline of hydrogen production within the first 20 cycles is suggested to be due to the growth of a silicon dioxide and zircon layer which acts as a diffusion barrier for the reacting specie.

## 1. Introduction

The production of hydrogen without deploying fossil resources is one of the main challenges that have to be overcome for building a future hydrogen economy. By using water as a resource and concentrated solar energy to provide the necessary process heat, hydrogen can be produced basically without CO_2_ formation. Because of thermodynamic restrictions, significant yields in the direct thermal splitting of water can only be achieved at temperatures above 2500 K. Although temperatures that high can be reached with solar concentrating systems, they still impose extraordinary demands on materials and reactor design. Thermochemical cycles split water in several steps and enable hydrogen generation at moderate temperatures, which are manageable by today’s technical equipment. Another major advantage is that hydrogen and oxygen are produced in separate steps, *i.e.*, no separation of hydrogen and oxygen is needed. A number of possible thermochemical cycles exist and have been reviewed over the last years [1,2,3]. Another option would be the electrolysis of water using the electricity generated by a solar power plant. Electrolysis is an already mature technology that is seen as a benchmark for future hydrogen production. Comparative studies that have been published showed that thermochemical cycles have the potential of a higher efficiency than electrolysis and hence the potential to reduce the production costs of hydrogen from water [4,5,6].

Among the most interesting thermochemical cycles are the two step water-splitting cycles using metal oxide redox-systems [7]. During the first step of this cycle (the regeneration step) the metal oxide is reduced by setting some of its lattice oxygen free according to Reaction (1).

In the next step (the water-splitting step) the reduced and thus activated material is oxidized by water vapor. This means that oxygen ions refill oxygen vacancies of the metal oxide and simultaneously hydrogen is produced (Reaction 2).

MO_oxidized_ → MO_reduced_ + O_2_ (1)

MO_reduced_ + H_2_O → MO_oxidized_ + H_2_ (2)

Several metal oxide processes have been evaluated, e.g., iron oxide Fe_3_O_4_/FeO [8,9,10,11,12], manganese oxide Mn_3_O_4_/MnO [13] and zinc oxide ZnO/Zn [14]. The high temperatures (2100 K) needed for the reduction of magnetite Fe_3_O_4_ lead to rapid deactivation of the material. The necessary reduction temperature can be lowered by supporting Fe_3_O_4_ on yttria-stabilized zirconia (YSZ), for which a new reaction mechanism was proposed [15,16]. Mixed-metal ferrites, which incorporate transition metals in the ferrite structure, can be reduced at lower reduction temperatures and are being studied intensively. Such transition metals are manganese (Mn) [17,18], cobalt (Co) [19], nickel (Ni) [20,21] and zinc (Zn) [22,23,24]. Several comparative studies have already been published [25,26].

Different approaches on solar receiver concepts using mixed ferrites [27,28,29,30,31,32,33] have been developed. One of those approaches incorporates honeycomb structures that are coated with the active material and placed in a solar receiver-reactor where they act on the one hand as the absorber for the concentrated solar irradiation and on the other hand provide the necessary surface area for the chemical reaction. The advantages of such a concept are its simplicity, as it has no moving parts, and its scalability. Such coated monoliths had shown the capability of water splitting and regeneration at moderate temperatures of 1200 °C. However, a strong deactivation of the material was observed with on-going testing [34,35].

First studies with coated SiSiC monoliths were presented in a previous work showing the influence of water splitting temperature and water concentration on the hydrogen production rate [34]. The work showed that the ferrite coating sintered strongly during the high temperature regeneration step leading to a loss of porosity and surface area. The occurrence of unintended reactions between the ferrite and the SiSiC substrate was suggested but not finally resolved. In the present work, coated SiSiC honeycomb structures with a zinc ferrite redox material were analyzed with different material characterization techniques after they were cycled in a laboratory test set-up in order to resolve any interactions between the substrate and the coating.

## 2. Experimental Set-Up

**Figure 1:**
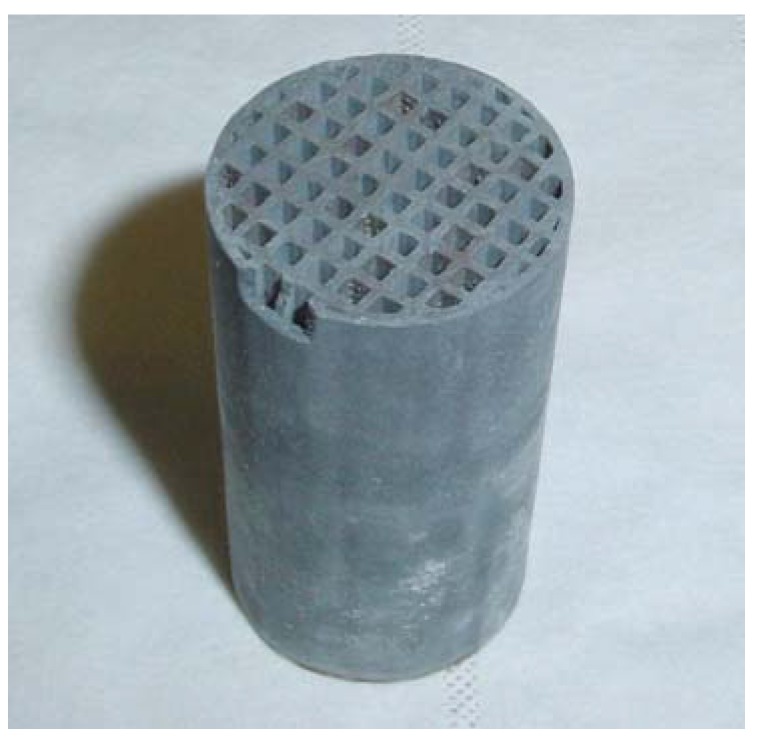
Siliconized silicon carbide (SiSiC) honeycomb structure used for testing.

As in the previous work the tested honeycomb structures shown in Figure 1 (outer diameter: 26 mm; length: 50 mm; channel density: 90 channels per square inch) consisted of a substrate of SiSiC coated with a zinc ferrite of the form Zn*_x_*Fe_3−*x*_O_4_. The SiSiC honeycombs were coated with the zinc ferrite via a washcoating technique employed for the coating of automotive catalysts [36]. During the washcoating procedure the porous supports were impregnated with a slurry containing the coating powder and aluminum oxide (Al_2_O_3_) as a binding material. The honeycombs were then dried and fired at 800 °C under an inert gas atmosphere. To prevent any reactions between the redox powder and the support material, a barrier coating layer of zircon dioxide (ZrO_2_) was applied on the monolith to offer structural and chemical stabilization. After the impregnation the honeycombs were fired to ensure efficient adhesion of the redox material. With this method the honeycomb structures were coated with a thin layer of the redox material. The coverage was complete but the thickness of the coating varied strongly as will be shown in the following paragraph.

The laboratory test set-up in which the samples were tested was already described in the previous work [34]. In the test set-up, a coated honeycomb can be exposed to changing gas atmospheres of either pure inert gas or a mixture of water-vapor and inert gas while it is heated by an electric furnace. In this way the sample undergoes alternately the reduction and the water-splitting step. Hydrogen production was monitored with a mass spectrometer in the off-gas line of the reactor. Excess water was condensed in a gas cooler before the mass spectrometer.

For the present work, the coated honeycomb structures were subjected to different numbers of cycles, one cycle comprising of one water-splitting and one high-temperature regeneration step. For water-splitting, the samples were subjected to a mixture of water vapor and nitrogen for about 20 min. Then the water vapor was switched off and the temperature was increased to 1200 °C in order to regenerate the redox material. During this step and during heating and cooling phases the sample is always flushed with a constant nitrogen stream. After testing, the coated honeycombs were investigated using different methods for material characterization in order to show material transformations and the formation of possible reaction products between the coating and the SiSiC substrate. For the analysis, small pieces were broken off the monoliths after testing. For phase analysis the samples were subjected to X-ray diffractometry (XRD) with CuK_α_ radiation (Siemens, Kristalloflex D5000 diffractometer). Analysis of the X-ray diffraction pattern in order to identify the phases was done with the software tool EVA2 (Bruker) by comparing the diffraction data of the analysed sample against powder diffraction file (pdf) database. The microstructure of the samples was characterized by scanning electron microscopy (Zeiss, Ultra55, cathode FEG) equipped with an energy dispersive X-ray spectroscopy (EDS) unit (Oxford) for microanalysis. For Scanning Electron Microscopy (SEM)-EDS analysis the samples were fixed on small sample holders using conductive silver and/or conductive foil. In order to prevent charging effects, SEM samples were coated with platinum. To gain information on the elemental distribution of a sample, small pieces of the honeycomb or powder samples were embedded in epoxy resin to prepare polished cross-sections, which were then examined by SEM-EDS.

## 3. Experimental Results

The ferrite loading of all samples tested and the number of cycles they had performed are summarized in Table 1. Also water splitting and regeneration temperatures are shown. With Monolith 3 and 4 parametric studies concerning the effect of water splitting temperature on the hydrogen production rate were performed. The temperature was kept constant during a water-splitting step but varied from cycle to cycle. Temperatures between 800 and 1200 °C were investigated. With Monolith 5 parametric studies concerning the regeneration temperature were performed varying the temperature between 900 and 1200 °C. After testing, all samples were analyzed via XRD and SEM-EDS analysis as described above. The XRD patterns of the samples are displayed in Appendix A.

**Table 1:** Overview of investigated coated honeycomb structures.

Sample notation	Ferrite loading per gram of coated monolith	Total ferrite loading	Treatment
Monolith 1	0.071 g/g_monolith_	2.30 g	untreated
Monolith 2	0.070 g/g_monolith_	2.27 g	1 cycle water splitting at 900 °C regeneration at 1200 °C
Monolith 3	0.104 g/g_monolith_	3.48 g	25 cycles water splitting between 800 and 1200 °C regeneration at 1200 °C
Monolith 4	0.169 g/g_monolith_	6.12 g	58 cycles water splitting between 800 and 1200 °C regeneration at 1200 °C
Monolith 5	0.100 g/g_monolith_	3.35 g	74 cycles water splitting at 900 °C regeneration between 900 and 1210 °C

As shown in the previous work, typically hydrogen production started with a peak at a high level as soon as water vapor was introduced into the reactor but then decreased rapidly within about 10 min to a small but constant level [34]. No oxygen could be detected in the off-gas of the reactor in all the experiments during the regeneration phase. The release and presence of oxygen in the gas phases would be expected by Reaction (1). It was suggested that the released oxygen reacts with the substrate forming silicon dioxide SiO_2_. This will now be verified by comparing the untreated honeycomb (Monolith 1) to the treated honeycombs (Monoliths 2–5).

**Figure 2:**
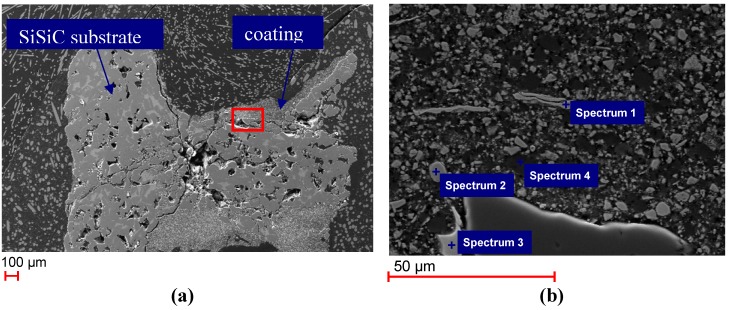
(**a**) Scanning Electron Microscopy (SEM) images of polished cross section of Monolith 1 (untreated); (**b**) enlarged cutout of Figure 2a showing the coating of Monolith 1 and points where energy dispersive X-ray spectroscopy (EDS) were taken.

SEM images of the polished cross section of the unused Monolith 1 are shown in Figure 2. The coating is distributed quite inhomogeneously on the substrate, which becomes clear in Figure 2a with varying coating thickness between 50 and 300 μm. The encircled area in Figure 2a is shown as an enlarged cutout in Figure 2b.

EDS analysis taken at different locations on the sample (compare Figure 2b revealed various elemental compositions (Table 2). On the one hand, pure iron particles (Spectrum 1) and a zinc and iron containing phase (Spectrum 2) originating from the zinc ferrite did occur. On the other hand, isolated light-colored Zr-bearing crystals were detected (Spectrum 3), which originate from the ZrO_2_ barrier coating. The Zn-ferrite, Fe and ZrO_2_ grains were embedded in alumina (Spectrum 4), which was used in the coating process as a binding material.

**Table 2:** Elements detected in Spectra 1–4 in Figure 2.

Spectrum	Elements detected
Spectrum 1	Fe
Spectrum 2	Fe, O, Zn
Spectrum 3	O, Zr
Spectrum 4	Al, O

**Figure 3:**
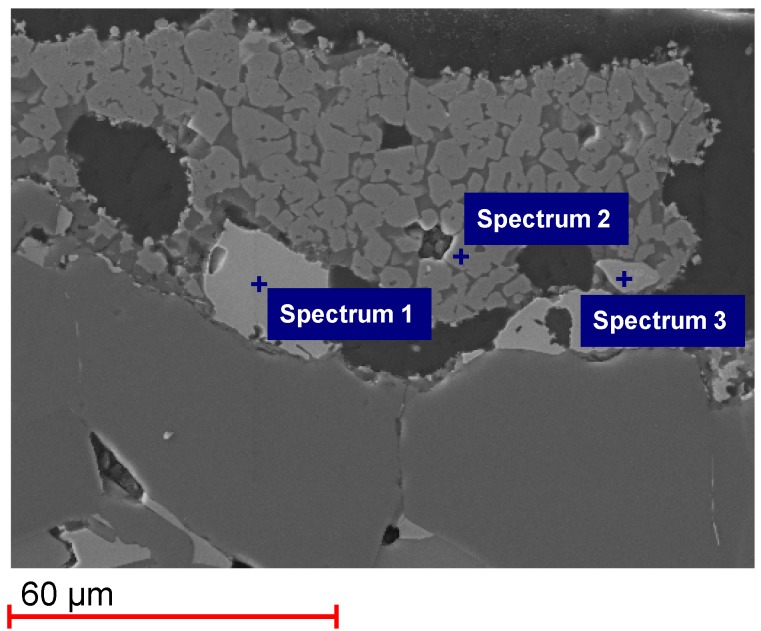
SEM image and EDS spectra of Monolith 2 after 1 cycle.

Figure 3 shows an SEM image of Monolith 2, tested for only one cycle. The elements detected in the EDS spectra are shown in Table 3. The XRD pattern of Monolith 2 is shown in Appendix A. A Fe-Si phase could be found in the SEM-EDS analysis (Spectrum 1), which was identified by XRD analysis to be FeSi. A Fe-Al-O phase was detected (Spectrum 2), which is probably hercynite FeAl_2_O_4_. It was not found in the XRD pattern probably because it is below the detection limit of XRD analysis. Finally, a Zr-Si-O phase was found in the SEM-EDS analysis (Spectrum 3), which has been identified in the XRD pattern to be zircon (ZrSiO_4_).

**Table 3:** Elements detected in Spectra 1–3 in Figure 3.

Spectrum	Elements detected
Spectrum 1	Fe, Si
Spectrum 2	Al, Fe, O
Spectrum 3	O, Si, Zr

SEM-EDS and XRD analysis of Monoliths 3, 4 and 5 also showed the existence of these new phases; especially FeSi and ZrSiO_4_. As well the XRD analysis of all the tested monoliths (Monoliths 2–5) showed clearly the existence of crystalline SiO_2_ (cristobalite or tridymite; respectively; see Appendix A). The presence of these phases shows that multiple reactions have occurred between the different phases present in the coating and the SiSiC substrate. Comparing the peaks heights of the new phases for all monoliths in the XRD patterns (Appendix A); an increase of peak height with increasing cycle number could be observed; suggesting that the reaction products are formed throughout repeated cycling.

**Figure 4:**
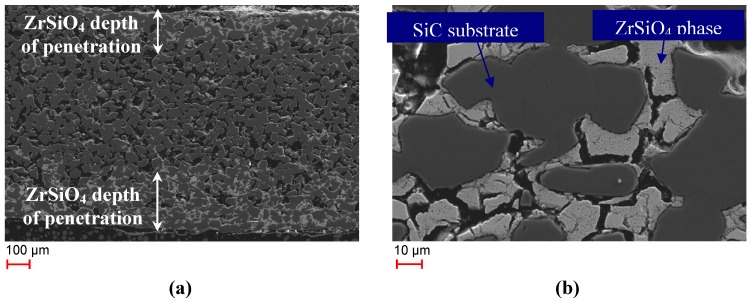
(**a**) SEM image of polished cross-section of Monolith 4 after 58 cycles (**b**) enlarged cutout of Figure 4a.

Another interesting observation was made when looking at the cross sections of the SiSiC substrate. The SEM images displayed in Figure 4 shows a section through a SiSiC bar of Monolith 4 that had been tested for 58 cycles in the laboratory test-rig.

In Figure 4a a light-colored phase that has penetrated about 150–250 µm into the monolith on both sides is visible. SEM-EDS analysis showed that the phase contains Zr, Si and O, *i.e.*, it is the zircon phase detected in the XRD patterns. The ZrSiO_4_ phase encloses the darker SiC grains (Figure 4b). A thin layer of SiO_2_ was detected by SEM-EDS at the contact sites of zircon and the SiC grains. Scattered inclusions of pure ZrO_2_ were detected in the ZrSiO_4_ phase. The course of formation of the zircon phase inside the substrate is not fully clear though the following scenario appears feasible: First, a silica scale has formed through oxidation of Si and SiC. In the next step adjacent ZrO_2_ grains might have been dissolved in the amorphous silica thus reducing its viscosity. Then the viscous zirconia-silicate phase infiltrated the porous SiSiC ceramics and, after approaching a certain composition, ZrSiO_4_ has been formed. SEM-EDS analysis of Monoliths 3 and 5 also showed the existence of a ZrSiO_4_ phase that had penetrated into the monoliths. Table 4 shows the thickness of the ZrSiO_4_ layer for the different monoliths. The layer of Monolith 4 was thickest which might be due to the combination of high numbers of cycles performed and high temperatures up to 1200 °C.

**Table 4:** Thickness of ZrSiO_4_ phase.

Sample	Thickness of ZrSiO_4_ phase
Monolith 3	30 µm
Monolith 4	150–250 µm
Monolith 5	120 µm

Another finding was that in the inner region of Monolith 4 no free Si is present anymore in contrast to the untreated monoliths where Si is found between the SiC grains. Instead, EDS analyses of the substrate bulk material of Monolith 4 indicated a silica phase that had formed around the inner SiC grains as can be seen in the EDS mapping of Monolith 4 in Appendix B. The observations suggest that free Si has completely reacted, forming first SiO_2_ and then other reaction products. The SiC grains have only oxidized on the edge, forming a protective SiO_2_ surface layer. The XRD data of Monolith 4 and 5 confirmed that no Si is present anymore in the sample while in the XRD data of Monoliths 1, 2 and 3 (tested for 0, 1, 25 cycles respectively) a Si peak was still observed. This observation is in correspondence with the findings of Mehan *et al.* who investigated the reactions of Si/SiC (polycrystalline SiC crystals immersed in a matrix of silicon, the material consisted of 70% SiC, 25% Si and 5% C) with metals (Ni, Co, Cr) and metal alloys at temperatures up to 1150 °C [37]. They found that the composite material was depleted of silicon, leaving voids between the converted fiber bundles. Complex mixed silicides had formed in the reacted metal, leading them to conclude that Si had diffused directly into the metallic or alloy phase. Experiments with pure SiC and Si_3_N_4_ ceramics showed that the reaction was much less pronounced than in presence of silicon-containing materials. They concluded that in the case of SiC and Si_3_N_4_, much of the surface had been rendered inert by a SiO_2_ layer. Their results correspond well to the observations of the present study. The substrate of Monolith 4 was also depleted of Si, suggesting that Si had completely reacted with the coating material.

## 4. Discussion

The SEM-EDS and the XRD analyses showed clearly that a number of reactions between the SiSiC substrate, the Zn-ferrite coating, the ZrO_2_ barrier coating and the Al_2_O_3_ binder material had occurred during testing of coated SiSiC honeycombs forming new reaction products such as FeSi, ZrSiO_4_ and SiO_2_. Especially the fact that no oxygen was detected in the off-gas of the reactor during the regeneration step must be due to the fact that the ferrite is reduced through reaction with Si and SiC, hence forming SiO_2_.

This leads to the formulation of a new reduction mechanism for the redox material through reaction with free Si and through reaction with SiC:

MO_oxidized_ + Si → MO_reduced_ + SiO_2_ (3)

MO_oxidized_ + SiC → MO_reduced_ + SiO_2_ + CO (4)

The formation of CO was not monitored during the tests described above because the mass spectrometer used to monitor the off-gas composition cannot distinguish between CO and N_2_, used as a carrier gas. Some tests were performed with Argon instead of N_2_ and in these CO formation was observed in the first couple of reduction steps, meaning that probably both reactions take place in the first cycles. The SiC surface is probably quickly rendered inert by a SiO_2_ layer forming on the outside of the SiC grains. Thus, we assume that the reduction of the ferrite occurs mainly through reaction with free Si as long as Si is present in the structure. It is suggested that the reduction of the ferrite deteriorates with cycling due to the growing of a SiO_2_ and ZrSiO_4_ layer and the disappearance of Si. Thus, reduction might be controlled by permeability and diffusion of silicon or oxygen through the product layer. This is supported when looking at the produced amount of hydrogen over many numbers of cycles.

**Figure 5:**
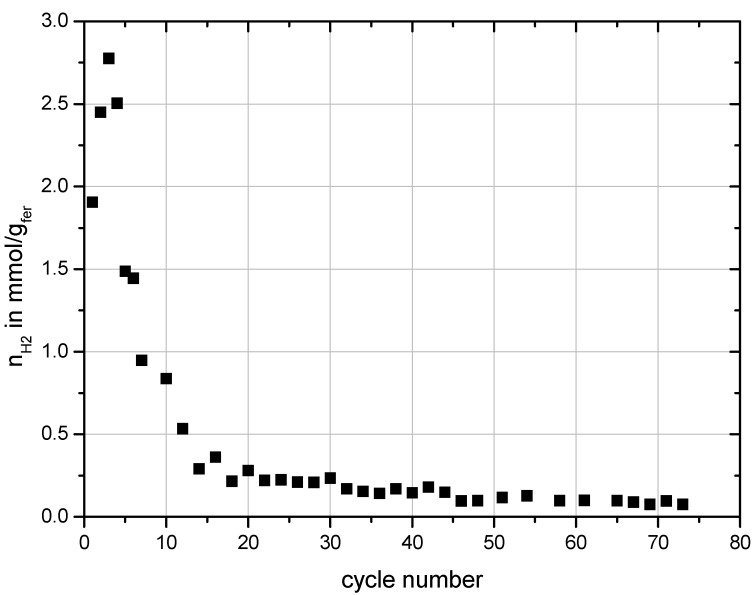
Specific hydrogen production over cycle number performed with Monolith 5.

Figure 5 shows the total specific amount of hydrogen produced during the water-splitting steps performed with Monolith 5. In these cycles water-splitting was always performed at 900 °C for 20 min with a constant flow rate of water of 0.59 mmol/s introduced into the reactor. The preceding regeneration step was always performed at 1200 °C. A decline of the hydrogen production especially within the first 20 cycles is observed. Then hydrogen production settled to an almost constant level and only slightly decreased with on-going testing.

As shown already in the previous work, comparison of SEM images of untreated and treated samples showed that the coating strongly sinters leading to a decrease of initial porosity and thus surface area [34]. But the SEM images of the monolith after one cycle (Monolith 2) showed that this effect already occurs in the first high temperature reduction step. That means any decrease in hydrogen production due to the loss of surface area should be seen from the first to the second cycle.

The results presented above showed that the substrate is slowly depleted of Si with on-going testing. After about 50 cycles no free Si could be found anymore in the honeycomb and the SiC grains of the substrate had formed a protective layer of SiO_2_. It is suggested that this is the reason for the decrease of the hydrogen production rate with on-going testing. As long as free Si is available the reduction proceeds fast but after about 20 cycles the reduction of the ferrite decreases to a very small level when the free Si in the substrate is completely converted to SiO_2_ resulting in the very low amounts of hydrogen production after the 20th cycle shown in Figure 5. At that point, reduction of the ferrite can occur to some extend through thermal reduction and through reaction with SiC by diffusion of oxygen through the SiO_2_ layer developed around the SiC grains. In none of the reduction steps any O_2_ was monitored in the off-gas of the reactor, meaning that if thermal reduction occurred, the O_2_ is either reacted on the monolith or somewhere downstream of the reactor. To what extend these two reactions take place cannot be resolved at this time.

## 5. Conclusions

SiSiC honeycomb structures coated with a zinc ferrite redox material were tested in a laboratory test set-up and analyzed afterwards through SEM-EDS and XRD analyses. The material characterization showed the formation of various reaction products after testing such as silicon dioxide, zircon, iron silicide and hercynite indicating the occurrence of various reactions between the different phases of the coating as well as between the coating and the SiSiC substrate. Comparison of several tested honeycombs showed that the Si content in the samples declined with repeated cycling whereas the amount of reaction products increased. Combining this with the observation that no oxygen is released during the regeneration led us to the conclusion that the ferrite is reduced through reaction with Si and SiC. A decline of the hydrogen production was observed especially within the first 20 cycles. The growing of a silicon dioxide and zircon layer, which acts as diffusion barrier for Si and oxygen was suggested as a main reason for the decline of hydrogen production. After about 25 cycles most of the free Si had reacted and reduction could proceed very slowly through diffusion of oxygen through the oxide layer and through reaction with SiC or through thermal reduction, thus leading to very small hydrogen production rates.

We have seen that with the investigated structures consisting of a ferrite on a SiSiC substrate, the SiSiC enabled the reduction of the ferrite at moderate temperatures of 1200 °C. Temperatures that low have not been reached yet with pure ferrite systems. But the deterioration of the hydrogen production due to the consumption of the Si and SiC is unavoidable leading to the conclusion that the system in its current state is not feasible to use in a water-splitting cycle. A way to prevent reactions between the support and the coating would be either to use an effective barrier coating or a different substrate than SiSiC or even to abandon the substrate completely by producing structures completely made out of the reactive material.

## Figures and Tables

**Figure A1:**
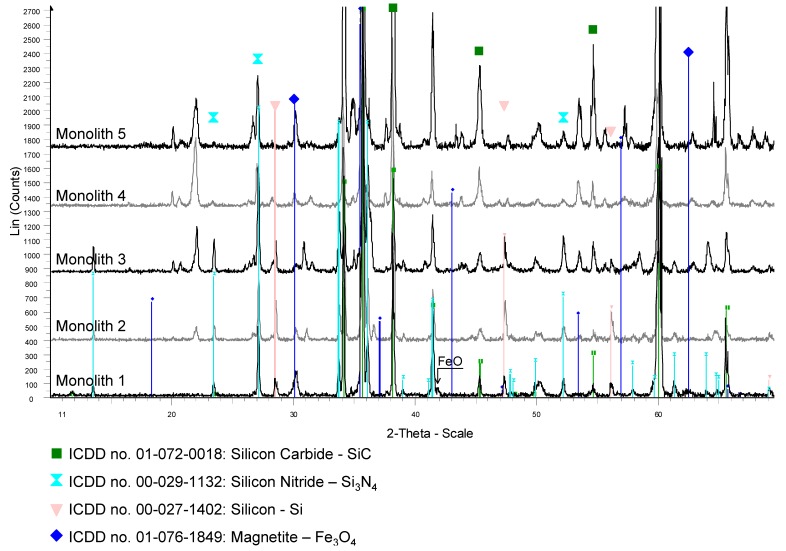
X-ray diffractometry (XRD) patterns of coated SiSiC honeycombs, phases shown are SiC, Si_3_N_4_, Si and Fe_3_O_4_.

**Figure A2:**
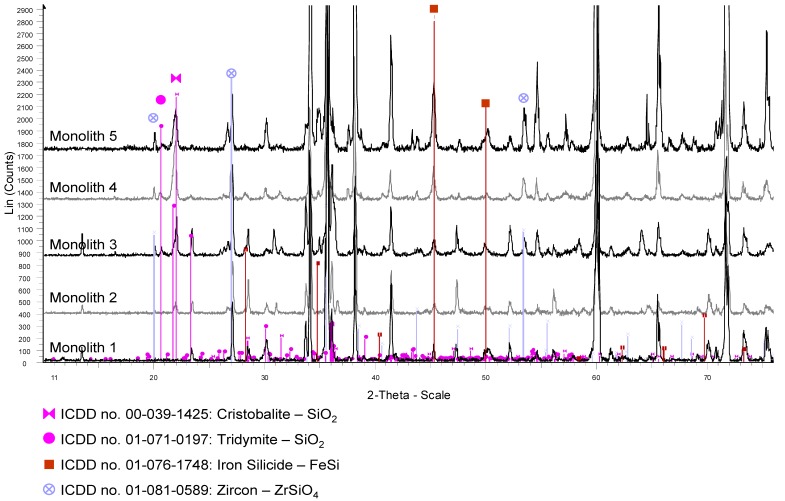
XRD patterns of coated SiSiC honeycombs, phases shown are SiO_2_, FeSi and ZrSiO_4_.

**Figure B2:**
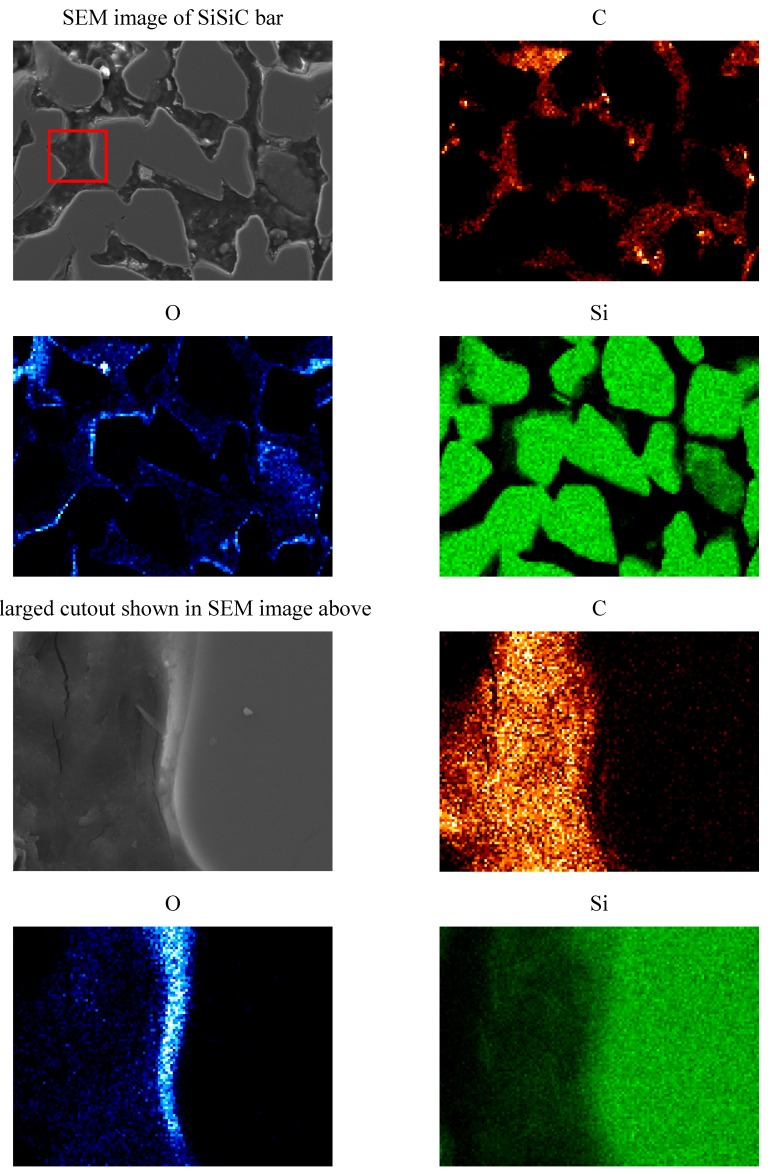
energy dispersive X-ray spectroscopy (EDS) mapping of Monolith 4.
